# Clinical characteristics of single human papillomavirus 53 infection: a retrospective study of 419 cases

**DOI:** 10.1186/s12879-021-06853-7

**Published:** 2021-11-15

**Authors:** Ruizhe Chen, Yunfeng Fu, Bingbing You, Ying Li, Yeli Yao, Xinyu Wang, Xiaodong Cheng

**Affiliations:** Women’s Hospital, Zhejiang University School of Medicine, Hangzhou, 310006 People’s Republic of China

**Keywords:** Human papillomavirus, Cervical intraepithelial neoplasia, Single infection, Genotype

## Abstract

**Background:**

Human papillomavirus (HPV) infection is the main cause of cervical cancer. Characteristics of HPV infections, including the HPV genotype and duration of infection, determine a patient’s risk of high-grade lesions. Risk quantification of cervical lesions caused by different HPV genotypes is an important component of evaluation of cervical lesion. Data and evidence are necessary to gain a deeper understanding of the pathogenicity of different HPV genotypes. The present study investigated the clinical characteristics of patients infected with single human papillomavirus (HPV) 53.

**Methods:**

This retrospective study analyzed the clinical data of patients who underwent cervical colposcopy guided biopsy between October 2015 and January 2021. The clinical outcomes and the follow-up results of the patients with single HPV53 infection were described.

**Results:**

82.3% of the initial histological results of all 419 patients with single HPV53 infection showed negative (Neg). The number of patients with cervical intraepithelial neoplasia (CIN)1, CIN2, CIN3, vaginal intraepithelial neoplasia (VaIN)1, CIN1 + VaIN1, CIN1 + VaIN2, and CIN2 + VaIN2 was 45, 10, 2, 9, 6, 1, and 1, respectively. Cancer was not detected in any patient. When the cytology was negative for intraepithelial lesion or malignancy (NILM), atypical squamous cells of undetermined significance (ASC-US) or low-grade squamous intraepithelial lesion (LSIL), we observed a significant difference in the distribution of histological results (*P* < 0.05). 95 patients underwent follow-up with cytology according to the exclusion criteria. No progression of high-grade lesions was observed during the follow-up period of 3–34 months.

**Conclusions:**

The lesion caused by HPV53 infection progressed slowly. The pathogenicity of a single HPV53 infection was low.

## Background

Cervical cancer is the fourth most frequently diagnosed cancer and the fourth leading cause of cancer death in women, with an estimated 604,000 new cases and 342,000 deaths worldwide in 2020 [1]. The incidence and mortality rates of this malignancy have declined in most areas of the world over the past few decades; however, mortality rates for cervical cancers are considerably higher in developing vs. developed countries (12.4 vs. 5.2 per 100,000) [1]. The disease burden of cervical cancer is significantly high in China, the world’s most populous developing country. Statistical data show that in 2018, an estimated 106,430 new cases and 47,739 deaths in China were attributable to cervical cancer [2]. The incidence rate of cervical cancer has increased significantly, and the mortality rate is also on the rise [3].

Human papillomavirus (HPV) infection is the main cause of cervical cancer [4, 5]. Based on frequency of occurrence in cases of cervical cancer and available biological data, apha HPV types are classified as “carcinogenic to humans” (The International Agency for Research on Cancer [IARC] classification Group 1), “probably/possibly carcinogenic to humans” (IARC Groups 2A and 2B), and “not classifiable as to its carcinogenicity to humans” (IARC Group 3) [6]. The HPV53 genotype belongs to the IARC Group 2B and is relatively common in the population worldwide. So et al. [7] analyzed 1988 samples from healthy women, as well as those from women with cervical intraepithelial neoplasia (CIN) 1–3 and cervical cancer, and observed that HPV53 (9.3%) was the fourth most common HPV genotype among all samples in the study. Reportedly, HPV53 was one of the most frequent genotypes detected by several studies (prevalence rate 4.1–9.69%) [8–10]. Additionally, although HPV53 was considered to be a possible carcinogenic HPV type, it was detected in < 0.5% of invasive cervical cancer [11, 12]. Although many studies have focused on HPV infection, the clinical characteristics and risks of HPV53 infection remain inconclusive.

This study retrospectively analyzed the clinical data of patients who underwent cervical colposcopy guided biopsy. We investigated the clinical outcomes and follow-up results in patients with single HPV53 infection and recommend evidence-based clinical intervention strategies.

## Methods

### Study design and participants

**Fig. 1:**
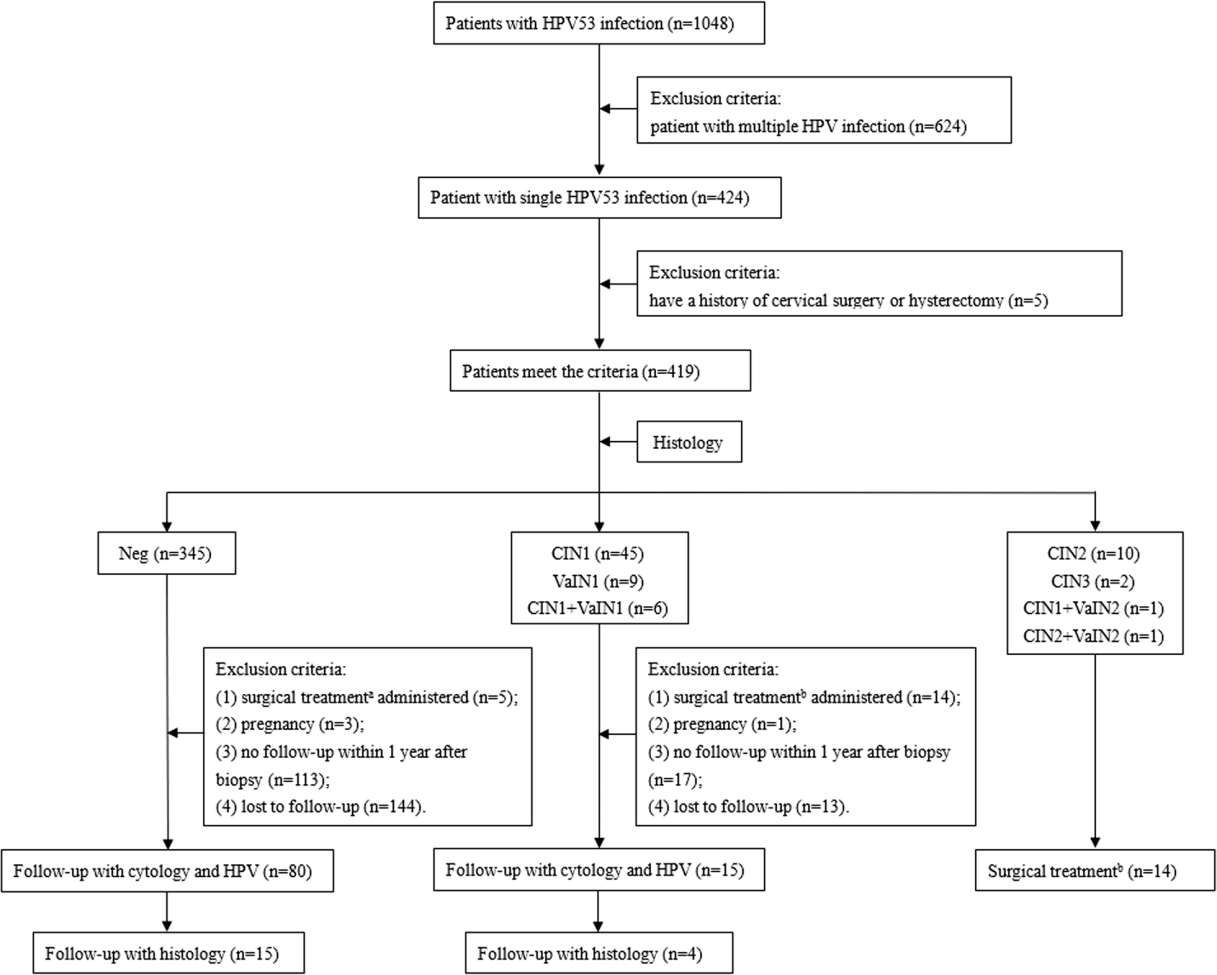
Study flowchart. *Notes* ^a^Underwent hysterectomy for other indications; ^b^Surgical treatment includes excision and ablation therapy. *HPV* human papillomavirus; *Neg* negative; *CIN* cervical intraepithelial neoplasia; *VaIN* vaginal intraepithelial neoplasia

This retrospective case–control study was conducted in the Women’s Hospital, Zhejiang University School of Medicine, China, and was approved by the Institutional Ethics Committee (PRO2021-1292). We analyzed the clinical data of patients who underwent cervical colposcopy guided biopsy between October 2015 and January 2021. Among the 1048 patients with HPV53 infection, 424(40.5%) had single HPV53 infection and 624 (59.5%) had multiple HPV infection. Among the 424 patients, 419 patients without a history of cervical operation or hysterectomy were enrolled in the final statistical analysis (Fig. 1).

Following were the exclusion criteria: (1) surgical treatment administered, (2) pregnancy, (3) no follow-up within 1 year after biopsy, and (4) lost to follow-up. By January 2021, 95 of 419 patients underwent follow-up for 3–34 months after colposcopy guided biopsy. All patients underwent cytological evaluation (examination of cervicovaginal exfoliated cells) and HPV testing during follow-up. Some patients with new indications for colposcopy guided biopsy underwent new histological analysis. In addition to cervical examination, we performed careful colposcopy evaluation of the upper third of the vagina, including the fornices in all patients. Patients who underwent surgical treatment including excision and ablation therapy showed negative results on cytological evaluation performed at initial follow-up.

### Standards and classification

Patient age, the type of HPV infection, cytological and histological findings, and follow-up data were obtained from medical records. HPV genotyping was performed using polymerase chain reaction-based technology, including 13 high-risk HPV genotypes (HPV 16, 18, 31, 33, 35, 39, 45, 51, 52, 56, 58, 59, and 68) and 2 possible high-risk HPV genotypes (HPV 53 and 66) [13]. Cytological findings were categorized as follows based on the 2001 Bethesda classification system: negative for intraepithelial lesion or malignancy (NILM), atypical squamous cells of undetermined significance (ASC-US), low-grade squamous intraepithelial lesion (LSIL), atypical squamous cells cannot exclude high-grade squamous intraepithelial lesion (ASC-H), atypical glandular cells (AGC) (including subcategories of AGC), high-grade squamous intraepithelial lesion (HSIL), and inadequate [14]. Histologic findings were interpreted based on the worst result obtained among all colposcopy guided biopsies, and these were categorized as follows: negative (Neg), CIN 1–3, and vaginal intraepithelial neoplasia (VaIN) 1–3.

### Statistical analysis

All statistical analyses were performed using the Statistical Package for the Social Sciences Version 21.0 (SPSS Inc., Chicago, IL, USA). Continuous non-normally distributed variables are presented as median (range). Categorical variables are expressed as frequencies and percentages. The (One-sample) Chi-square test were used whenever appropriate. Statistical tests were 2-sided, and *P* values < 0.05 were considered statistically significant.

## Results

Patients’ median age was 45 (range 21–70) years. The number of patients with CIN1, CIN2, CIN3, VaIN1, CIN1 + VaIN1, CIN1 + VaIN2, and CIN2 + VaIN2 was 45, 10, 2, 9, 6, 1, and 1, respectively. Table 1 shows histological findings corresponding to the cytological results. Cancer was not detected in any patient in this study. When the cytology showed NILM, ASC-US and LSIL, we observed a significant difference in the distribution of histological results (*P* < 0.05).

**Table 1:** Distribution of histology corresponding to different cytology

Cytology	Histology								*P*
	Neg	CIN1	CIN2	CIN3	VaIN1	CIN1 + VaIN1	CIN1 + VaIN2	CIN2 + VaIN2	
NILM (n = 199)	182(91.5%)	9(4.5%)	3(1.5%)	–	3(1.5%)	2(1.0%)	–	–	< 0.001
ASC-US (n = 111)	93(83.8%)	13(11.7%)	4(3.6%)	–	–	1(0.9%)	–	–	< 0.001
LSIL (n = 97)	66(68.0%)	19(19.6%)	–	2(2.1%)	6(6.2%)	3(3.1%)	1(1.0%)	–	< 0.001
ASC-H (n = 6)	2(33.3%)	2(33.3%)	2(33.3%)	–	–	–	–	–	1.000
HSIL (n = 2)	–	–	1(50.0%)	–	–	–	–	1(50.0%)	1.000
Unknow (n = 4)	2(50.0%)	2(50.0%)	-	–	–	–	–	–	1.000

*Neg* negative; *CIN* cervical intraepithelial neoplasia; *VaIN* vaginal intraepithelial neoplasia; *NILM* negative for intraepithelial lesion or malignancy; *ASC-US* atypical squamous cells of undetermined significance; *LSIL* low-grade squamous intraepithelial lesion; *ASC-H* atypical squamous cells cannot exclude high-grade squamous intraepithelial lesion; *HSIL* high-grade squamous intraepithelial lesion

Based on the aforementioned exclusion criteria, 95 patients underwent follow-up with cytology. 19 of the 95 patients underwent colposcopy guided biopsy. The median follow-up period was 12 (range 3–34) months. The initial cytological results showed NILM (n = 49), ASC-US (n = 22), LSIL (n = 23) and ASC-H (n = 1). Follow-up cytological results showed NILM (n = 83), ASC-US (n = 6), LSIL (n = 5) and AGC (n = 1). Initial histological findings showed 80 (84.2%) patients had Neg, 8 (8.4%) had were CIN1, 5 (5.3%) had VaIN1, and 2 (2.1%) had CIN1 + VaIN1 lesions. Follow-up histology showed results, 17 (89.5%) patients had Neg, 1 (5.3%) had CIN1, and 1 (5.3%) had VaIN1 lesions. During follow-up, 26 patients were infected with other high-risk HPV types (Table 2). No progression of high-grade lesions was observed during follow-up.

**Table 2:** Follow-up outcomes of patients with a single HPV53 infection

Initial cytology (n = 95)	Initial histology (n = 95)	Follow-up cytology (n = 95)	Follow-up histology (n = 19)	Other high risk HPV infection during follow-up (n = 26)
NILM (49)	Neg (45)	NILM (44)	Neg (6)	9
		ASC-US (1)	Neg (1)	
	CIN1 (1)	NILM (1)		
	VAIN1 (2)	NILM (2)		
	CIN1 + VaIN1 (1)	NILM (1)		1
ASC-US (22)	Neg (20)	NILM (14)		4
		ASC-US (2)	Neg (2)	1
		LSIL (3)	Neg (3)	2
		AGC (1)	Neg (1)	1
	CIN1 (2)	NILM (1)		
		ASC-US (1)	Neg (1)	1
LSIL (23)	Neg (14)	NILM (13)		2
		LSIL (1)	CIN1 (1)	1
	CIN1 (5)	NILM (4)	Neg (1)	1
		ASC-US (1)	Neg (1)	
	VaIN1 (3)	NILM (2)		1
		LSIL (1)	VaIN1 (1)	1
	CIN1 + VaIN1 (1)	NILM (1)		1
ASC-H (1)	Neg (1)	ASC-US (1)	Neg (1)	

(49): numbers between parenthesis refer to absolute numbers of cases with corresponding cytology or histology

*NILM* negative for intraepithelial lesion or malignancy; *Neg* negative; *ASC-US* atypical squamous cells of undetermined significance; *CIN* cervical intraepithelial neoplasia; *VaIN* vaginal intraepithelial neoplasia; *LSIL* low-grade squamous intraepithelial lesion; *AGC* atypical glandular cells; *ASC-H* atypical squamous cells cannot exclude high-grade squamous intraepithelial lesion

## Discussion

In this study, we investigated 419 patients without a history of cervical surgery or hysterectomy, who presented with single HPV53 infection. The pathogenicity of single HPV53 infection was low; 82.3% of the initial histological results showed Neg and 14.3% showed CIN1, VaIN1, or CIN1 + VaIN1 lesions. No progression of high-grade lesions was observed during the follow-up period of 3–34 months; therefore, it is reasonable to conclude that the lesions induced by single HPV53 infection progress slowly.

Characteristics of HPV infections, including the HPV genotype and duration of infection, determine a patient’s risk of high-grade lesions [15, 16]. Risk quantification of cervical lesions caused by different HPV genotypes is an important component of evaluation of cervical lesion [17]. Further data and evidence are necessary to gain a deeper understanding of the pathogenicity of different HPV genotypes. Owing to regional cultural differences, a large number of resources and expenditure are allocated for HPV53 screening in some areas, and a few reports suggest that HPV53 infection is relatively common [7, 8, 10, 18], which may lead to panic among the population regarding this infection. Literature review yielded a few articles that describe HPV53 infection and follow-up [19, 20]; however, most studies included a small number of patients or did not perform comparison of histological results. Our study investigated the clinical outcomes of HPV53 infection and provides evidence to support for the association between HPV53 infection and cervical lesions. The long follow-up period of 34 months described the developmental trend of HPV53 infection, which will contribute to inform clinicians of appropriate treatment and follow-up strategies. Furthermore, unnecessary treatment can be avoided and patients’ anxiety can be reduced.

A large number of epidemiological studies have shown significant differences in HPV infection status and distribution across different countries or regions worldwide. Del et al. [9] found that in Italy, HPV42 was the most prevalent virus type, followed by HPV16, 53 and 31, with a lower prevalence of the HPV11, 82 and 35 genotypes. Kantathavorn et al. [21] observed that HPV52, 16, and 51 were the most common high-risk HPV genotypes detected in Thai women and HPV52 was common in Asians. HPV 16, 18, 35 and 45 were the most prevalent genotypes in northeastern Brazil [22]. In China, HPV types are also different owing to their geographical distribution [13, 23–25]. HPV53 infection has been reported in various places, and is relatively common [8–10, 18]. We investigated more than 1000 patients with HPV53 infection, who were treated at single center between October 2015 and January 2021, which indicates that HPV53 infection was relatively common in the population. Moreover, reportedly, a high percentage of single HPV infections are associated with cervical lesion (66.7–91.5%) [26]. Based on the methods used for HPV detection, the prevalence of multiple infections ranged from 1 to 52% [9, 27, 28]. Dickson et al. [29] observed that the HPV53 genotype was more likely to occur in multiple infections with other genotypes. Our study results are consistent with these findings. Among the 1048 patients with HPV53 infection who underwent initial investigation, in addition to 424 (40.5%) patients with single HPV53 infection, we observed a significantly large percentage of 624 (59.5%) patients with multiple HPV infections.

Many studies have reported that in contrast to the HPV16 variety, which is the most common HPV genotype that causes invasive cervical carcinoma (55.2%) and high-grade lesions (45.1%) [2], the HPV53 genotype is more commonly associated with low-grade lesions [7, 30]. Based on data provided by Padalko et al. [20], a sevenfold difference is observed in the frequency of ASC-US/LSIL (82.4%) and HSIL + (11.8%) in the cytological results of HPV53 infection. However, a meta-analysis showed that different distribution of HPV genotypes may be detected in HIV-positive women with HSIL, who were significantly more likely to be infected with the HPV53 genotype [31]. Our study showed that 405(96.7%) patients with single HPV53 infection had histological results of Neg, CIN1, VaIN1, or CIN1 + VaIN1, and only 14 (3.3%) patients showed CIN2, CIN3, CIN1 + VaIN2, or CIN2 + VaIN2. No patient showed cancer in our study, and no patient showed progression of high-grade lesions during follow-up. In our view, although the HPV53 genotype is designated as “possibly high risk” variety, the pathogenicity of single HPV53 infection was not so serious, and it is not a rapidly progressive infection.

The main limitation of the study is its retrospective nature. Although all data were obtained from medical records, due to the quality of cytological specimens and the variety of HPV genotyping methods, samples and test results were obtained from multiple centers, and discrepancies in findings across various centers may have affected our results. Currently, no reference standard is available for HPV genotyping, and further research is warranted in this field. Notably, in this study, the cytological findings were classified based on the 2001 Bethesda classification system, and histological results of colposcopy guided biopsy were obtained from a single center. Therefore, in our opinion, our conclusions are reliable. As mentioned earlier, this was a single-center study, and multicenter studies are warranted to exclude possible biases associated with single-center research.

## Conclusions

Our study indicates that single HPV53 infection shows low pathogenicity and is not a rapidly progressive condition. Combined with the results of cytological screening, some patients with single HPV53 infection might appropriately extend the screening interval. Our findings contribute to inform clinicians of appropriate treatment and follow-up strategies for patients with single HPV53 infection.

## Data Availability

All relevant data are within the paper. Requests for additional information should be addressed to the corresponding author and data may be provided on reasonable request.

## Abbreviations

AGC: Atypical glandular cells
ASC-H: Atypical squamous cells cannot exclude high-grade squamous intraepithelial lesion
ASC-US: Atypical squamous cells of undetermined significance
CIN: Cervical intraepithelial neoplasia
HPV: Human papillomavirus
HSIL: High-grade squamous intraepithelial lesion
LSIL: Low-grade squamous intraepithelial lesion
Neg: Negative
NILM: Negative for intraepithelial lesion or malignancy
PCR: Polymerase chain reaction
SPSS: Statistical Package for the Social Sciences
VaIN: Vaginal intraepithelial neoplasia
